# Bacterial Translocation in Gastrointestinal Cancers and Cancer Treatment

**DOI:** 10.3390/biomedicines10020380

**Published:** 2022-02-04

**Authors:** Keita Kouzu, Hironori Tsujimoto, Yoji Kishi, Hideki Ueno, Nariyoshi Shinomiya

**Affiliations:** 1 Department of Surgery, National Defense Medical College, Tokorozawa 359-0042, Japan; kouzu24@ndmc.ac.jp (K.K.); ykishi-3su@ndmc.ac.jp (Y.K.); ueno_surg1@ndmc.ac.jp (H.U.); 2 National Defense Medical College, Tokorozawa 359-0042, Japan; shinomi@ndmc.ac.jp

**Keywords:** bacterial translocation, gastrointestinal cancer, synbiotics, pathogen-associated molecular patterns

## Abstract

In recent years, there has been increasing evidence that gut microbiota is associated with the onset and exacerbation of various diseases, such as gastrointestinal cancer. For instance, it is well known that local inflammation of the intestinal tract in colorectal cancer that is caused by the increased number of *Fusobacterium*, due to changes in the intestinal bacterial flora, is involved in carcinogenesis. In contrast, gut bacteria or their products, pathogen-associated molecular patterns, not only cause intestinal inflammation but also invade the bloodstream through dysbiosis and gut barrier dysfunction, thereby leading to systemic inflammation, namely bacterial translocation. The involvement of bacterial translocation in the carcinogenesis of gastrointestinal cancers and their prognosis is increasingly being recognized. The Toll-like receptor signaling pathways plays an important role in the carcinogenesis of such cancers. In addition, bacterial translocation influences the treatment of cancers such as surgery and chemotherapy. In this review, we outline the concept of bacterial translocation, summarize the current knowledge on the relationship between gut bacteria and gastrointestinal cancer, and provide future perspectives of this field.

## 1. Introduction

In recent years, many gut bacterial species and metabolites correlated with carcinogenesis have been identified. Several studies have also reported about the involvement of gut bacteria in carcinogenesis outside the gastrointestinal tract, including the hematopoietic system [1,2]. In humans, the highest abundance of commensal bacteria is found in the colon [3]. Among all the gut bacteria, those belonging to the genus *Fusobacterium* have been extensively studied for their possible association with colorectal cancer [4]. The transplantation of *Fusarium nucleatum* into mice alters the tumor microenvironment and activates E-cadherin and β-catenin signaling, which leads to the regulation of the oncogenic response [1]. Patients with colon cancer who have a high amount of *F. nucleatum* have been reported to have a poorer prognosis [5]. 

**Figure 1:**
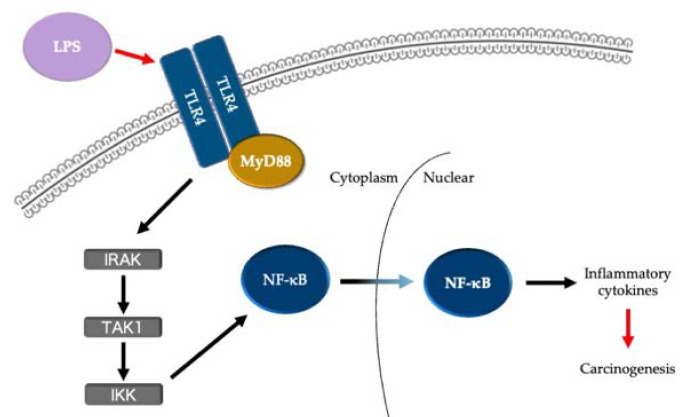
TLR4/MyD88/NF-κB pathway involved in the carcinogenesis of colorectal cancer. LPS, lipopolysaccharide; TLR, Toll-like receptor; IRAK, IL-1 receptor associated kinase; TAK1, transforming growth factor β activated kinase 1; IKK, IκB kinase.

The relationship between gut microbe-induced inflammation and carcinogenesis has recently emerged as a topic of interest. For instance, lipopolysaccharides (LPSs) are reportedly involved in the carcinogenesis of colorectal cancer through the activation of nuclear factor kappa B (NF-κB) and β-catenin via Toll-like receptor (TLR) 4 in the gut, which is predominantly colonized by gram-negative bacilli [6,7]. In other words, LPS, as a typical example of pathogen-associated molecular patterns (PAMPs), and TLR4, as a pattern recognition receptor (PRR), can initiate signaling pathways that subsequently activate a series of immune and inflammatory responses in the host that are induced by microbial infection as well as carcinogenesis (Figure 1) [8,9].

The association between local inflammation of the gastrointestinal tract and gastrointestinal cancer is adequately established; moreover, bacterial translocation, a type of systemic inflammation caused by gut bacteria, has now been shown to be associated with gastrointestinal cancer. The concept of bacterial translocation was defined by Berg et al. as “the passage of viable bacteria from the gastrointestinal tract through the epithelial mucosa into the lamina propria and then to the mesenteric lymph nodes and possible other organs” [10]. Gastrointestinal cancer itself is also a cause of bacterial translocation, and it is important to prevent and treat bacterial translocation to terminate the negative feedback loop between bacterial translocation and gastrointestinal cancer. In addition, systemic infections caused by bacterial translocation can contribute to a delay in cancer treatment.

In this review, we have summarized the current knowledge on bacterial translocation and gastrointestinal cancer, including treatment strategies for bacterial translocation. We have also provided an outlook on the desired future treatments.

## 2. Search Strategy

In this study, the PubMed/Medline database was searched for articles published from 1974 to 2021. The search terms were as follows: “bacterial translocation” and “cancer (or carcinoma).” From 266 manuscripts searched in the literature database, 64 were selected for this review. Any literature on oral bacteria and cancers other than gastrointestinal cancer was excluded. As their contents were not applicable to this review, 193 manuscripts were excluded. In addition, nine manuscripts were excluded because they were not written in English. Furthermore, the articles cited in the references of the selected articles were reviewed.

## 3. Pathogen-Associated Molecular Patterns

The intestinal tract is one of the largest immune organs in the body with gut-associated lymphoid tissue and contains lymphocytes, plasma cells, and macrophages, which produce mediators such as cytokines. In the past, the clinical diagnosis of bacterial translocation required culture detection of enteric bacteria from the bloodstream and mesenteric lymph nodes. However, even if bacteria are not identified in the blood or tissues, systemic inflammation may be triggered via the production of mediators from the intestinal tract. There are two main types of stimulants that are involved in the initiation of events in inflammation: PAMPs, which are components of bacteria and viruses; and damage-associated molecular patterns (DAMPs), such as high-mobility group box chromosomal protein 1, which are released by damaged cells and the extracellular matrix [11]. 

In recent years, the concept of bacterial translocation has evolved to include not only the translocation of bacteria and endotoxins but also the translocation of PAMPs and DAMPs [12,13]. Indeed, microbial-specific DNA has been detected using polymerase chain reaction (PCR) techniques in the blood of patients after highly invasive surgeries, such as hepatectomy and esophagectomy, and such techniques were shown to be more sensitive than blood culture for providing confirmation of bacterial translocation [14,15]. Therefore, the concept of bacterial translocation is changing; previously, it was considered to relate to the entry of enteric bacteria into the bloodstream and tissues. However, this may be interpreted as triggering a systemic inflammatory response via intestinal immune cells by gut bacteria. This change, which expands the concept of bacterial translocation to include PAMPs and DAMPs, may aid in understanding the pathogenesis of some cases that have troubled clinicians in the past, wherein bacterial translocation was suspected but could not be diagnosed because the blood cultures were negative.

## 4. Pattern Recognition Receptors

**Figure 2:**
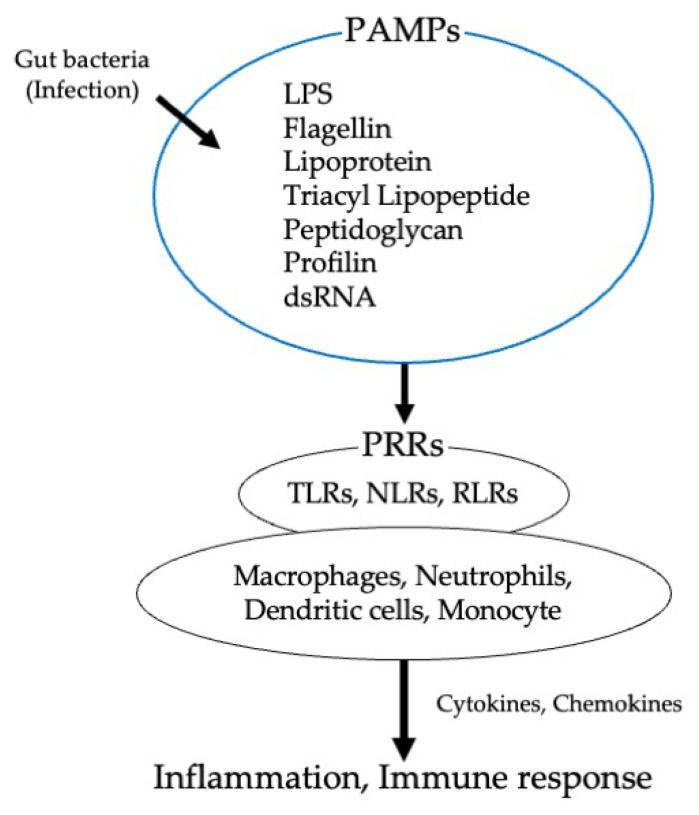
Mechanisms by which gut bacteria induce inflammatory and immune responses via PAMPs and PRRs. PAMPs, pattern recognition receptors; LPS, lipopolysaccharide; PRRs; pattern recognition receptors; TLRs, Toll-like receptors; NLR, NOD-like receptors; RLR, RIG-I-like receptors.

Shared receptors, including membrane-bound or endosomal PRRs, have been identified for PAMPs and DAMPs. PRRs can detect the presence of bacterial products and trigger intracellular signaling cascades that result in an inflammatory response [16]. PRRs include TLRs, nucleotide-binding oligomerization domain (NOD)-like receptors, retinoic acid-inducible gene I (RIG-I)-like receptors (RLRs), and C-type lectin receptors. PRRs are expressed by various types of immune cells such as macrophages, neutrophils, monocytes, and dendritic cells (Figure 2).

Immune cells use the TLRs expressed on the cell surface to recognize bacteria. There are 10 functional TLRs in humans (TLR1–10) and each TLR recognizes and binds to a different PAMP as a ligand. Because gut bacteria are composed of LPS, peptidoglycan, flagellin, and CpG DNA, it is believed that they are recognized by immune cells via TLRs. Peptidoglycan is recognized by TLR2, LPS is recognized by TLR4, flagellin is recognized by TLR5, and CpG DNA is recognized by TLR9. Table 1 presents the exogenous and endogenous ligands of TLRs (Table 1). Mice lacking myeloid differentiation primary-response protein 88 (MyD88), a downstream signaling molecule of TLRs, not only present with low levels of intestinal IgA antibody but also exhibit reduced mucus acidity, intestinal epithelial cell proliferation, and antimicrobial substance production. This indicates the importance of the recognition of gut bacteria by TLRs [17].

TLR2 recognizes the most diverse range of ligands among all TLRs and responds not only to PAMPs but also to DAMPs. This broad response is attributed to the fact that TLR2 forms a heterodimer with TLR1 and TLR6. The TLR2 heterodimer induces an intracellular signaling cascade that leads to the MyD88-dependent activation of activator protein-1 and NF-κB and the formation of inflammasomes [18]. TLR4 is a receptor for gram-negative LPS and its main PAMP lipid A. In response to LPS, TLR4 interacts with three different extracellular proteins: LPS-binding protein, cluster of differentiation (CD)14, and myeloid differentiation protein 2. TLR4 signaling involves two pathways: the MyD88-dependent pathway and the MyD88-independent pathway. In the MyD88-independent pathway, the Toll-interleukin-1 receptor domain-containing adaptor inducing the interferon (IFN)-β-related adaptor molecule leads to IFN-β production and IFN-inducible gene expression via the activation of IFN-regulatory factor 3 [19].

The cell surface receptor TLR5 forms homodimers and recognizes flagellin, a major component of bacterial flagellar filaments, in both gram-positive and gram-negative bacteria. TLR5 activation induces the production of proinflammatory cytokines through signal transduction via MyD88. Depending on the cell type, TLR5 and flagellin elicit different innate immune responses. The TLR5–flagellin interaction induces the production of high levels of IL-8 in epithelial cells but causes the secretion of proinflammatory cytokines, such as tumor necrosis factor (TNF), in monocytes and dendritic cells [20,21]. TLR9 is expressed within the endosomal compartments and recognizes a specific unmethylated CpG-oligodeoxynucleotide sequence that distinguishes microbial DNA from mammalian DNA. Similar to many other TLRs, TLR9 signaling is MyD88-dependent and acts on the transcription factors activator protein-1, NF-κB, and IFN-regulatory factor 7 [22].

With the advancement of immunological research, many new adjuvants have been developed. In particular, the usefulness of TLR ligands as therapeutic medicine has already been investigated in many clinical trials. For instance, monophosphoryl lipid A, a TLR4 ligand, has been used as a vaccine adjuvant for cervical cancer. In addition, imiquimod, a TLR7 agonist, is effective against basal cell carcinoma. TLRs may contribute to cancer therapeutics or vaccine adjuvants [38,39].

Recent findings indicate an association with gastrointestinal cancers in other PRRs as well. The NOD-like receptor family recognizes non-self signals, activates NF-κB, and binds to caspase-1, resulting in the formation of a complex known as the inflammasome. Inflammasomes activate proinflammatory cytokine signaling and are involved in maintaining gut microbiota [40,41]. NF-κB plays a central role in NLR-mediated inflammation and carcinogenesis of gastrointestinal cancers, particularly in inflammatory bowel disease-related colorectal cancer [42,43].

Unlike TLRs, RIG-I-like receptors recognize virus-derived non-self RNA in the cytoplasm and induce the production of IFNs and proinflammatory cytokines [44]. A member of the RIG-I-like receptors, RIG-I, has attracted attention for its antitumor activity. RIG-I-deficient mice were reported to be susceptible to colitis-associated colorectal cancer, with reduced IgA and regenerating islet-derived protein 3 gamma levels, and disrupted intestinal microbiota [45]. In addition, Poly(I:C), which is an agonist RLR, induces apoptotic signaling pathways in human gastric adenocarcinoma cells [46].

C-type lectin-like receptors have various functions, such as cell adhesion, phagocytosis, complement activation, and innate immune responses, and recognize DAMPs and PAMPs on the surface of fungi, bacteria, and viruses [47]. Lectin-like oxidized low density lipoprotein receptor-1, one of the C-type lectin-like receptors, promotes the migration and invasion of gastric cancer cells and is overexpressed in human colon cancers [48,49].

## 5. Bacterial Translocation and Carcinogenesis

As mentioned in the introduction, evidence on the relationship between intestinal bacteria and the carcinogenesis of gastrointestinal cancers is beginning to accumulate. However, in the carcinogenesis of gastrointestinal cancers in which intestinal bacteria are indigenous, showing the extent to which bacterial translocation is involved is challenging. Many studies have been conducted on gut microbiota and cancer of the liver and pancreas, which is an organ located outside the gastrointestinal tract. Unlike gastrointestinal cancers, which are driven by changes in the local environment that result from dysbiosis and chronic inflammation, liver and pancreatic cancers are more likely to be associated with bacterial translocation.

The liver is an organ that receives portal blood flow from the gastrointestinal tract, and the relationship between chronic liver disease and gut bacteria has been adequately characterized. A metagenomic analysis revealed that chronic liver disease was associated with dysbiosis of the gut microbiota [50]. A previous report showed that knockout mice lacking functional TLR-4 and sterilized with non-absorbable antibiotics in the intestinal tract have reduced levels of adiposity, oxidative stress, and liver inflammation [51]. The development of hepatocellular carcinoma (HCC) is closely associated with chronic inflammation, including hepatitis C virus infection and the consumption of alcohol. In addition, patients with nonalcoholic fatty liver disease, a liver disease that precedes HCC, have been shown to have higher levels of LPS and LPS-binding protein [52]. 

It has been suggested that LPS and flagellin cause inflammation and oxidative stress in the liver and promote HCC in animals [53,54]. Similarly, flagellin in high doses activates TLR5 signaling, which is involved in inflammation and oxidative stress, and contributes to the progression and severity of liver injury [55]. Furthermore, it was reported that the epithelial-to-mesenchymal transition in HCC cells could be induced by LPS [56]. In addition, a positive association between the response of IgA and IgG antibodies to LPS and flagellin and the risk of HCC in humans has been determined from multicenter studies [57,58]. In other words, the disruption of gut microbiota has the ability to promote the growth of endotoxin-producing bacteria and a leaky gut, ultimately transferring bacteria and bacterial metabolites to the liver, and inducing liver damage and carcinogenesis via the activation of the TLR-4 and NF-κB pathways [59,60].

In the study of pancreatic cancer, the presence of bacterial flora, including *Fusobacterium*, has been demonstrated in the tissues of patients with pancreatic ductal adenocarcinoma (PDAC), although most of the normal pancreatic tissue was found to be sterile [61]. Survival after PDAC surgery can be correlated with the composition of the tumor microbiome [62]. Bacterial dysbiosis associated with PDAC reportedly results in the suppression of both innate and adaptive immune systems [63]. The underlying mechanism for this was believed to be that PAMPs from gut bacteria activate TLR2 and TLR5 in tumor-associated macrophages, inducing a decrease in CD8+ cells and an increase in myeloid-derived suppressor cells. In addition, it was reported that fungi migrating from the gut lumen to the pancreas were involved in the pathogenesis of PDAC [64]. The ablation of the mycobiome has been shown to inhibit the progression of PDAC. In particular, *Malassezia* species are strongly involved in the progression of PDAC; their transfer from the intestinal tract into tumors is believed to cause the activation of complement C3 via a mannose-binding lectin, which binds to the glycans of the fungal wall, leading to tumor growth.

The discovery of the involvement of gut bacteria in cancer of the pancreas, which was believed to be a sterile organ, has been instrumental in identifying the relationship between bacterial translocation and carcinogenesis, which is now expected to be examined from the perspective of the relationship between carcinogenesis prevention and gut microbiota.

It is difficult to directly and clearly ascertain whether gastrointestinal cancer is caused by local inflammation or the bacterial translocation of gut microbiota. However, in patients with colorectal cancer, distant metastasis has been shown to be considerably more common in patients with high serum level of procalcitonin. [65] Procalcitonin is expressed by bacterial pathogens in multiple organs and is a clinically important biomarker to indicate the presence of bacterial infection [66]. Some reports have shown that procalcitonin and IL-6 are associated with the liver metastasis of cancer [67]. It is not surprising that bacterial translocation is involved in the hematogenous metastasis of gastrointestinal cancers, just as bacterial translocation is involved in the development of liver and pancreatic cancers. Since distant metastasis of cancer is a major determinant of patient prognosis, further research in this area is needed.

## 6. Bacterial Translocation and Cancer Surgery, Perioperative Management

Although surgery is the first choice for curative treatment of gastrointestinal cancers, it is an invasive treatment. Complications associated with infection in the perioperative period of gastrointestinal cancers are known to lead to poor long-term prognosis [68]. Postoperative complications may also delay the initiation of adjuvant therapy and interfere with appropriate cancer treatment, leading to a poor prognosis.

A prospective observational study showed that subjecting mesenteric lymph node tissues from patients with aortic dissection to bacterial culture exhibited a considerably higher rate of infectious complications in the positive cases than in the negative cases, suggesting that surgical invasion itself can cause bacterial translocation [69]. Herein, we discuss bacterial translocation and its prevention in the perioperative period of radical gastrointestinal cancer surgery.

Patients who have undergone surgical invasion have been known to develop transient systemic inflammatory response syndrome (SIRS) after surgery. The duration of SIRS after gastrointestinal cancer surgery is known to be associated with long-term prognosis [70]. Serum levels of cytokines, such as TNFα, IL-1β, IL-6, IL-10, and IL-2R, are increased in SIRS, and among these, IL-6 in particular is known to be involved in cancer progression and C-reactive protein synthesis in the liver [71]. As a clinically convenient technique, C-reactive protein has also been widely reported as a biomarker for the estimation of the association between SIRS and the long-term prognosis of cancer [72]. The gut microbiota of patients with SIRS differed from those of healthy volunteers, namely *Bifidobacterium* and *Lactobacillus* were decreased, whereas pathogenic *Staphylococcus* and *Pseudomonas* were markedly increased, as shown in studies of patients with severe infections and trauma [73]. In the perioperative period, it has been shown that surgical invasion and fasting can increase intestinal permeability and induce gut barrier dysfunction [74]. In addition, direct evidence suggests that microbiome changes occur before and after gastrointestinal surgery. Clinical studies have shown a significant increase in *Pseudomonas*, *Enterococcus*, *Staphylococcus*, and Enterobacteriaceae after performing a resection of colon in patients with colorectal cancer [75]. In the perioperative period of gastric cancer surgery, the relative abundances of the genera *Akkermansia*, *Escherichia/Shigella*, *Lactobacillus*, and *Dialister* were considerably altered [76]. 

In summary, gastrointestinal cancer surgery provides an environment in which bacterial translocation can occur owing to the disruption of the intestinal barrier function and changes in the gut microbiota. After surgical manipulation of the bowel, bacteria that were not found immediately after laparotomy were detected in mesenteric lymph node samples [77]. Furthermore, the reverse transcription–quantitative PCR results of mesenteric lymph node tissues and blood revealed that bacterial translocation occurred in more than 50% of the patients after esophagectomy [78]. Based on these results, the analysis of trace amounts of bacteria in the blood contaminated during surgery may be useful for perioperative infection management [79].

An interesting comparison was made between laparoscopic surgeries, a minimally invasive procedure, and open surgery for colorectal cancer, but there was no significant difference in the incidence of postoperative bacterial translocation between the two procedures [80]. Although laparoscopic surgery is less invasive than open surgery, the decrease in portal blood flow owing to insufflation pressure may promote bacterial translocation by producing free oxygen radicals and inhibiting mucosal integrity [81]. The perioperative prevention of bacterial translocation may need to be comprehensive and extend beyond surgical techniques. Furthermore, the use of propofol as an intraoperative anesthetic may inhibit the activation of the NF-κB pathway by downregulating miR-155 expression and protect the intestinal barrier by reducing the production of inflammatory cytokines in mice [82].

In terms of prevention, the usefulness of probiotics in the perioperative period is a topic of interest in this field, and several randomized controlled trials have been conducted to this end. In particular, the perioperative administration of synbiotics has been shown to reduce infectious complications in highly invasive surgeries, including hepatectomy for cholangiocarcinoma and liver transplantation [83,84]. Another study showed that the administration of synbiotics after hepatectomy improved gut integrity in patients with cirrhosis [85]. Similarly, in the field of gastrointestinal surgery, the administration of synbiotics reduced postoperative infection and decreased the detection rate of bacteria in the mesenteric lymph node tissues and blood of patients after esophageal cancer surgery [86,87]. In gastric and colorectal cancer surgery, the administration of synbiotics was effective in preventing changes in the gut microbiota [88,89].

As bacterial translocation also worsens the prognosis of cancer, surgeons and other medical professionals involved in surgery should be aware of the risk of bacterial translocation induced by surgery and work towards preventing it.

## 7. Febrile Neutropenia

Systemic chemotherapy, including molecular targeted drugs and immune checkpoint inhibitors, is one of the most effective treatment modalities for unresectable or recurrent gastrointestinal cancer. In recent years, the relationship between the effects of chemotherapy and gut bacteria has been adequately studied. The oral administration of an antibiotic cocktail containing vancomycin, imipenem, and neomycin reportedly impairs the mice microbiota and the response to intratumorally injected CpG-oligonucleotide immunotherapy and platinum chemotherapy for subcutaneous tumor [90]. This is associated with the decreased production of inflammatory cytokines, such as TNF, by leukocytes in the tumor. CpG-oligonucleotide is the ligand for TLR9. The gut bacteria are also reportedly involved in the activation of cytotoxic T cells after chemotherapy [91]. However, some bacterial species have been reported to increase the efficacy of immune checkpoint inhibitors [92,93].

Immune checkpoint inhibitors are novel agents that regulate antitumor immune responses by suppressing T-cell activity. They have achieved remarkable clinical efficacy across multiple types of tumors. Immune checkpoint molecules are located in T cell membranes; cytotoxic T-lymphocyte–associated antigen 4 and programmed death (PD)-1 are representative. However, only a limited number of patients can benefit from this antitumor effect, and differences in gut microbiota may be used to predict the therapeutic effect. In one example, it was reported that *Akkermansia* *muciniphila* was more common in patients who responded to immune checkpoint inhibitors targeting the PD-1/programmed death-ligand 1 axis [93]. *A.* *muciniphila* is known to maintain the gut barrier mechanism, suppress inflammation, and improve the metabolic profile [94,95].

One of the most serious adverse events of chemotherapy is febrile neutropenia. Febrile neutropenia is a condition in which patients with abnormally low numbers of neutrophils (neutrophil count of <500 cells/mm^3^ in the blood) develop fever. Bacteremia is present in at least one-fifth of patients with this condition [96]. Bacterial translocation may occur alongside intestinal mucosal damage or the immune response to anticancer drugs and contribute to febrile neutropenia [97,98]. It is recommended that blood-culture tests for bacteria and fungi are immediately conducted in case of patients with febrile neutropenia.

However, the causative bacteria of febrile neutropenia are unknown in many clinical cases. rRNA-PCR analysis has confirmed the presence of bacteria in the blood of patients with cancer undergoing chemotherapy, who developed febrile neutropenia at a substantially higher rate than those without the bacteria [99]. This study also showed that bacterial translocation occurred in patients with or without chemotherapy. In addition, plasma endotoxin levels and soluble CD14, an indicator of the early host response to endotoxins, were higher in patients with febrile neutropenia [100]. Notably, the preventive administration of antimicrobial medicines was somewhat associated with increased plasma endotoxin levels. With regard to this association, Papanicolas et al. reported that the combination of chemotherapy and antimicrobial agents might increase the risk of infection of the bloodstream with multidrug-resistant bacteria [101]. Gut bacteria that exhibit resistance to antimicrobial agents may be predominant in the chemotherapy-altered intestinal microbiota and pass through the treatment-damaged intestinal mucosa into the bloodstream, exacerbating bacterial translocation.

While infections in patients with neutropenia can progress rapidly, infected patients cannot be reliably distinguished from non-infected patients at the time of presentation. Therefore, it is inevitable that broad-spectrum antibiotics will be employed to treat febrile neutropenia, and there are no current countermeasures to the emergence of resistant bacteria from this treatment. However, there have been attempts to prevent febrile neutropenia with probiotics [102]. In a phase II study of the probiotic strain *Enterococcus faecium* M-74 in patients with acute or chronic leukemia, no severe diarrhea was observed, and the tolerability of the probiotic therapy was excellent, but the primary endpoint of febrile neutropenia could not be prevented [103]. One of the reasons why febrile neutropenia could not be prevented was that patients with leukemia often had central venous catheters, which are a common route of infection for bacteria; indeed, the incidence of bacteremia caused by coagulase-negative staphylococci was high [103]. Several studies have shown that probiotics prevent gastrointestinal adverse events, such as diarrhea, during chemotherapy in many carcinomas, including leukemia, and it certainly appears that probiotics prevent gastrointestinal disorders associated with chemotherapy [104]. 

In contrast, in gastrointestinal cancers, promising results have been shown regarding the usefulness of synbiotics in preventing febrile neutropenia. In a randomized study of patients undergoing preoperative chemotherapy for esophageal cancer, the incidence of febrile neutropenia was significantly lower in the group of patients who were administered synbiotics for 2 days before chemotherapy compared with the control group [105]: *Bifidobacterium breve* strain Yakult, *Lactobacillus casei* strain Shirota, and galacto-oligosaccharides were used in synbiotics group and *Streptococcus faecalis* was used in control group. This study showed that synbiotics in addition to prebiotics were more useful for the prevention of febrile neutropenia than probiotics alone. Moreover, there may be suitable and unsuitable strains for preventing FN. The use of multiple strains may also be effective. Synbiotics may have more to offer, but it is important to know that they are reportedly not effective for improving intestinal permeability or the intestinal barrier [106,107].

Thus, bacterial translocation is also deeply involved in chemotherapy. Infectious adverse events may be fatal for patients undergoing chemotherapy and can also affect long-term prognosis by delaying cancer treatment and causing the deterioration of nutritional status. In other words, manipulating gut microbiota may improve the therapeutic outcome of cancer chemotherapy.

## 8. Future Perspective

The above summarizes the current knowledge of bacterial translocation and gastrointestinal cancers. Carcinogenesis involves the mechanisms of bacterial infection and the inflammatory response. In this context, PAMPs and TLRs contribute to a great extent. Invasive cancer treatments, such as surgery and chemotherapy, are risk factors for developing bacterial translocation. Bacterial translocation is an important issue for clinicians because it affects not only the acute phase of the disease but also the long-term prognosis of cancer. One of the essential elements to preventing bacterial translocation during cancer treatment is the use of synbiotics.

**Figure 3:**
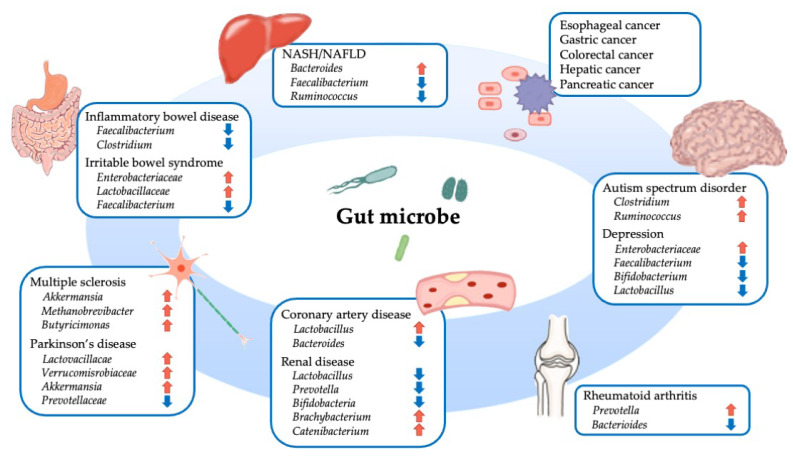
Overview of various diseases associated with gut microbes. NASH, nonalcoholic steatohepatitis; NAFLD, nonalcoholic fatty liver disease.

The gut microbiota has received much attention in recent years owing to its association with various diseases [108,109]. The gut microbiota is also involved in the pathogenesis of diseases that may not appear to be related to them at first glance (Figure 3). For instance, patients with rheumatoid arthritis, a progressive systemic autoimmune disease, reportedly showed an increased proportion of *Prevotella copri* and a decreased proportion of *Bacteroides fragilis* in their gut microflora [110]. In addition, this increase in *Prevotella* spp. was shown to occur in the pre-clinical stages of rheumatoid arthritis, suggesting that abnormalities in intestinal immunity are indicative of rheumatoid arthritis [111].

Furthermore, an association between gut microbiota and pathology has been noted in neurological and psychiatric disorders. Parkinson’s disease is a neurodegenerative disorder primarily characterized by the degeneration and loss of dopaminergic neurons associated with abnormal accumulation of α-synuclein in the substantia nigra. Although the causal relationship is not clear, gastrointestinal symptoms in Parkinson’s disease may precede motor symptoms, such as tremor, immobility, and muscle rigidity [112]. Multiple analyses using the 16S rRNA gene sequencing of microbial DNA or PCR of microbial DNA in stool samples have shown that alterations in the gut microbiota occur in patients with Parkinson’s disease; an increase in *Lactobacillaceae*, *Verrucomicrobiaceae*, *Lachnospiraceae*, and *Enterococcacea*, and a decrease in *Prevotellaceae* are common changes in many studies [113]. A clinical trial showed that probiotics ameliorated the symptoms of Parkinson’s disease [114].

Research has been ongoing on the brain–gut interaction and the microbiota–gut–brain axis in recent years, and evidence has accumulated to show that gut microbiota plays an important role in stress response and the etiology of mental disorders. The possibility of treatment with probiotics, prebiotics, or microbiota transfer therapy for patients with depression and patients with autism spectrum disorders is being explored [115,116].

Normally, the intestinal microbiota is adjusted and modified by genetic factors and environmental factors, such as diet, and is unique to each individual [117,118]. In terms of routinely used drugs, not only antibiotics but also non-steroidal anti-inflammatory and proton pump inhibitors have been known to affect the gut microbiota. Therefore, the intestinal microbiota can be acquired and changed, which represents a promising target for the treatment of diseases, including cancer. 

In contrast, the possibility of crosstalk owing to the coexistence of multiple microorganisms, rather than a single microorganism, is one of the complicating factors in this research. For instance, carcinogenesis by *Helicobacter pylori*, which known to be involved in gastric cancer, has been shown to be considerably inhibited by a lack of commensal flora in mice [119]. In addition to the development of new therapeutic methods, it is important to consider the possibility of reducing the risk of bacterial translocation-related carcinogenesis and resistance to cancer treatment by modifying the gut microbiota to a favorable state. It is hoped that research in this field will make more headway in the future.

## Figures and Tables

**Table 1:** Exogenous and endogenous ligands of Toll-like receptors (TLR) and main gastrointestinal cancers implicated in TLRs.

TLRs	Exogenous Ligands	Endogenous Ligands	Cancer	Citation
TLR1	Triacyl lipopeptide, LPS, Peptidoglycan	HSP, HMGB1, and Proteoglycans		
TLR 2	LPS, Peptidoglycan	HSP, HMGB1, Proteoglycans	GC, CRC	[23,24]
TLR 3	Double-stranded RNA	mRNA and tRNA	EAC, ESCC	[25,26]
TLR 4	LPS	Fibronectin, Polysaccharide fragments of heparan sulfate, HSP, Surfactant protein A in the lung epithelium 1, Neutrophil elastase, HMGB1, Biglycan	EAC, ESCC, GC, CRC	[27,28,29,30]
TLR 5	Flagellin		GC, CRC	[31,32]
TLR 6	Diacyl lipopeptide, Zymosan		CRC	[33]
TLR 7	Single-stranded RNA	Single-stranded RNA complex	EAC, ESCC, CRC	[25,34,35]
TLR 8	Single-stranded RNA, imidazoquinolines, guanosine analogs	Single-stranded RNA complex	EAC, CRC	[25,35]
TLR 9	Unmethylated CpG DNA	Chromatin–IgG complex	ESCC, GC, CRC	[34,36,37]
TLR 10	HIV-1 proteins			
TLR 11	Uropathogenic *Escherichia coli*			

LPS, lipopolysaccharide; HSP, heat shock proteins; HMGB1, high-mobility group protein 1; GC, gastric cancer; EAC, esophageal adenocarcinoma; ESCC, esophageal squamous cell carcinoma; CRC, colorectal cancer.

## Data Availability

Not applicable.
